# Improved Ammonia Synthesis and Energy Output from Zinc-Nitrate Batteries by Spin-State Regulation in Perovskite Oxides

**DOI:** 10.1021/jacs.4c12240

**Published:** 2025-01-16

**Authors:** Hele Guo, Yazhou Zhou, Kaibin Chu, Xueying Cao, Jingjing Qin, Nan Zhang, Maarten B. J. Roeffaers, Radek Zbořil, Johan Hofkens, Klaus Müllen, Feili Lai, Tianxi Liu

**Affiliations:** † The Key Laboratory of Synthetic and Biological Colloids, Ministry of Education, School of Chemical and Material Engineering, Jiangnan University, Wuxi 214122, China; ‡ Department of Chemistry, KU Leuven, Celestijnenlaan 200F, Leuven 3001, Belgium; § Max Planck Institute for Polymer Research, Ackermannweg 10, Mainz 55128, Germany; ∥ Nanotechnology Centre, Centre for Energy and Environmental Technologies (CEET), VŠB−Technical University of Ostrava, 17. listopadu 2172/15, Ostrava-Poruba 708 00, Czech Republic; ⊥ Key Laboratory for Colloid and Interface Chemistry, Ministry of Education, School of Chemistry and Chemical Engineering, Shandong University, Jinan 250100, P. R. China; # cMACS, Department of Microbial and Molecular Systems, KU Leuven, Celestijnenlaan 200F, Leuven 3001, Belgium; ¶ Regional Centre of Advanced Technologies and Materials, Czech Advanced Technology and Research Institute (CATRIN), Palacký University Olomouc, Šlechtitelů 241/27, Olomouc 779 00, Czech Republic; ∇ State Key Laboratory of Metal Matrix Composites, School of Materials Science and Engineering, Shanghai Jiao Tong University, Shanghai 200240, P. R. China

## Abstract

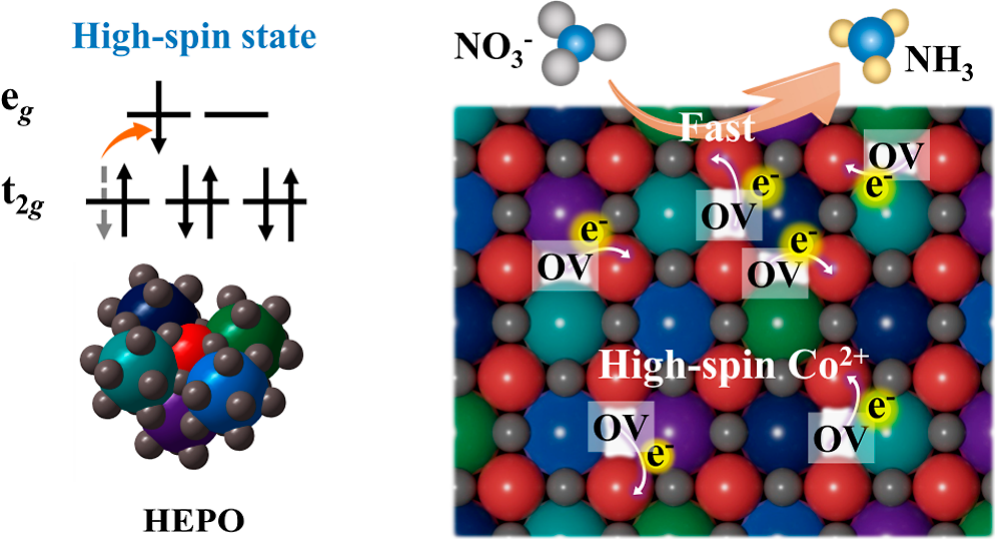

Electrocatalytic nitrate reduction to ammonia (eNRA) is a promising route toward environmental sustainability and clean energy. However, its efficiency is often limited by the slow conversion of intermediates due to spin-forbidden processes. Here, we introduce a novel A-site high-entropy strategy to develop a new perovskite oxide (La_0.2_Pr_0.2_Nd_0.2_Ba_0.2_Sr_0.2_)CoO_3-δ_ (LPNBSC) for eNRA. The LPNBSC possesses a higher concentration of high-spin (HS) cobalt-active centers, resulting from an increased concentration of [CoO_5_] structural motifs compared to conventional LaCoO_3_. Consequently, this material exhibits a significantly improved electrocatalytic performance toward ammonia (NH_3_) production, resulting in a 3-fold increase in yield rate (129 μmol h^–1^ mg_cat._^–1^) and a 2-fold increase in Faradaic efficiency (FE, 76%) compared to LaCoO_3_ at the optimal potential. Furthermore, the LPNBSC-based Zn-nitrate battery reaches a maximum FE of 82% and an NH_3_ yield rate of 57 μmol h^–1^ cm^–2^. Density functional theory calculations reveal that A-site high-entropy management in perovskites facilitates nitrate activation and potentially optimizes the thermodynamic rate-determining step of the eNRA process, namely, *HNO_3_ + H^+^ + e^–^ → *NO_2_ + H_2_O. This work presents an efficient concept for modulating the spin state of the B-site metal in perovskites and offers valuable insights for the design of high-performance eNRA catalysts.

## Introduction

Ammonia (NH_3_) is essential in modern society as a key feedstock for synthetic fertilizers and a carbon-free energy carrier.^1−3^ The global demand for NH_3_ exceeds 150 million tons annually, primarily produced by the energy-demanding Haber–Bosch process, which consumes 1%–2% of the world’s energy and contributes ∼1.4% to the global carbon emissions.^4,5^ Inspired by natural microbial nitrogen (N_2_) fixation, recent efforts have focused on the electrochemical reduction of N_2_ to NH_3_.^6,7^ However, the strong N≡N bond (941 kJ mol^–1^) and the poor solubility of N_2_ in water result in low NH_3_ yield rates and Faradaic efficiencies (FEs).^8,9^ The nitrate anion (NO_3_^–^) presents a more favorable alternative as a starting compound due to its lower N=O bond dissociation energy (204 kJ mol^–1^), thus holding promise for more favorable reaction kinetics in NH_3_ production.^10−13^ Additionally, NO_3_^–^ is prevalent in industrial wastewater and polluted groundwater, thereby leading to eutrophication and ecological disruptions.^14,15^ Therefore, the selective electrocatalytic nitrate reduction to ammonia (eNRA) presents an attractive solution to both the energy and environmental challenges.

The typical eNRA process involves the adsorption of NO_3_^–^ on the electrocatalytic surface, followed by a continuous deoxygenation process and subsequent stepwise hydrogenation to form NH_3_.^16−21^ Throughout this reaction sequence, the bonding interaction between the metal site and the intermediates is influenced by spin polarization.^22,23^ Spin-polarized metal-active sites can induce quantum spin exchange interactions and facilitate spin electron transfer, enhancing spin-dependent electrocatalytic reactions.^24−26^ However, accelerating the eNRA process by controlling spin states in electrocatalysts is challenging due to the complexity of its mechanism, which involves 8-electron-transfer steps, multiple intermediates, significant energy requirements, and sluggish kinetics. Currently, the spin state of cobalt (Co) species in the perovskite LaCoO_3_ has been investigated for its role in the oxygen evolution reaction (OER) and oxygen reduction reaction (ORR).^27,28^ The Co atoms of pristine LaCoO_3_ are in a low-spin (LS) state due to the unoccupied e_g_ electron orbitals.^29^ Occupation of the e_g_ orbitals implies a higher spin state for Co atoms, which can improve the adsorption of oxygen-containing species on the cations in octahedral sites.^29,30^ The spin state of Co cations in LaCoO_3_ can be effectively tuned using A/B site management strategies.^28^ A-site cation management helps to avoid the loss of active sites due to metal substitution, but it can cause stability issues due to cation segregation.^31,32^ Cation segregation happens when certain cations move and gather in specific areas, like surfaces or grain boundaries, in the perovskite structure.^33^ This can reduce the electrocatalytic activity and durability of the material, especially in conventional low-entropy perovskite oxides (LEPOs).^34^

High-entropy perovskite oxides (HEPOs) are a class of materials characterized by the incorporation of multiple principal cations, typically five or more at the A- and/or B-sites of the perovskite structure, resulting in high-configurational entropy.^35,36^ This unique composition imparts desirable properties, including lattice distortion, sluggish diffusion, and the “cocktail effect”, making HEPOs particularly attractive for applications in electrocatalysis.^35−37^ The diverse electronic structures and enhanced structural stability make HEPOs suitable for critical catalytic processes like ORR and OER, offering superior activity and durability compared to standard oxides.^35,38^ Remarkably enough, about 90% of metallic elements from the Periodic Table can theoretically be stabilized in perovskite oxides.^39^ This versatility allows HEPOs to fully leverage their structural and compositional advantages, thus opening up a vast potential for managing the spin states of core metals in perovskites and providing new avenues for developing high-performance eNRA catalysts.

**Figure 1:**
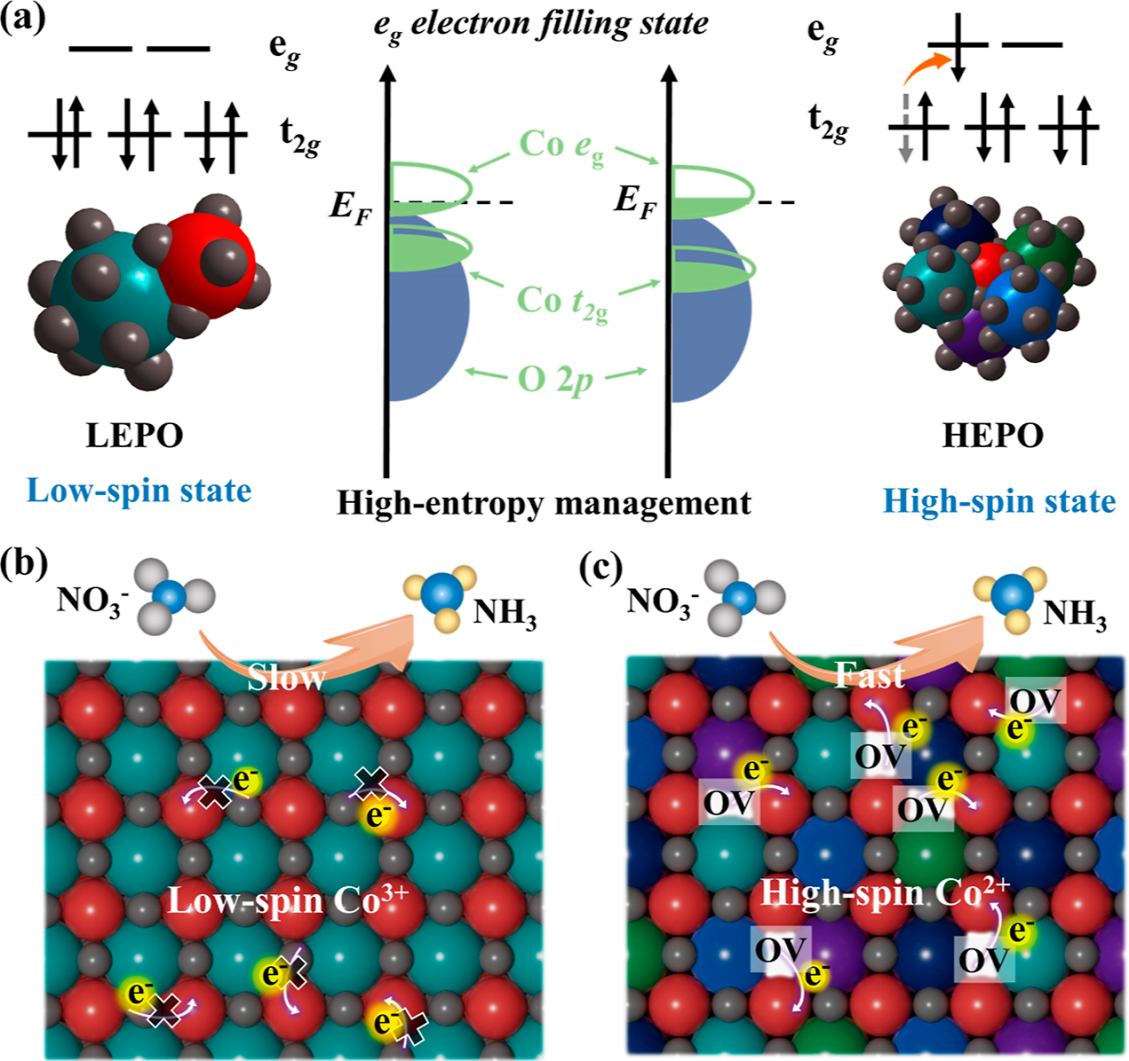
Schematic illustration of the (a) high-entropy management strategy for regulating the spin state of cobalt in perovskites, (b) slow eNRA process on the LEPO, and (c) fast eNRA process on the HEPO.

Inspired by the theory that e_g_ filling significantly influences the binding strength of oxygen-containing intermediates on the B-site active sites in perovskite oxides, we propose an A-site high-entropy management strategy to regulate the spin state of Co. This strategy effectively transforms Co from an LS state to a high-spin (HS) state, resulting in the reoccupancy of electrons in the e_g_ and t_2g_ orbitals (Figure 1a). Specifically, in LEPO, Co-active sites in the LS configuration exhibit a weaker activation effect on NO_3_^–^, leading to a sluggish eNRA process (Figure 1b). By contrast, in HEPO, Co-active sites in the HS state possess more unpaired electrons in the half-filled 3d orbital, which facilitates the electron transfer into the antibonding π*-orbital of NO_3_^–^, thereby enhancing the NO_3_^–^ activation (Figure 1c). Thus, the eNRA performance of the (La_0.2_Pr_0.2_Nd_0.2_Ba_0.2_Sr_0.2_)CoO_3–δ_ (LPNBSC) HEPO demonstrates substantially high NH_3_ yield rate and FE, with improvements by factors of approximately 3 and 2 times of LaCoO_3_ at the optimal potential, respectively. The underlying eNRA mechanism was further investigated by using a combination of online differential electrochemical mass spectrometry (DEMS) and density functional theory (DFT) calculations. The A-site high-entropy management effectively reduces the thermodynamic energy barrier of the rate-determining deoxygenation step (*HNO_3_ + H^+^ + e^–^ → *NO_2_ + H_2_O), thereby favoring and accelerating the overall eNRA process.

## Results and Discussion

**Figure 2:**
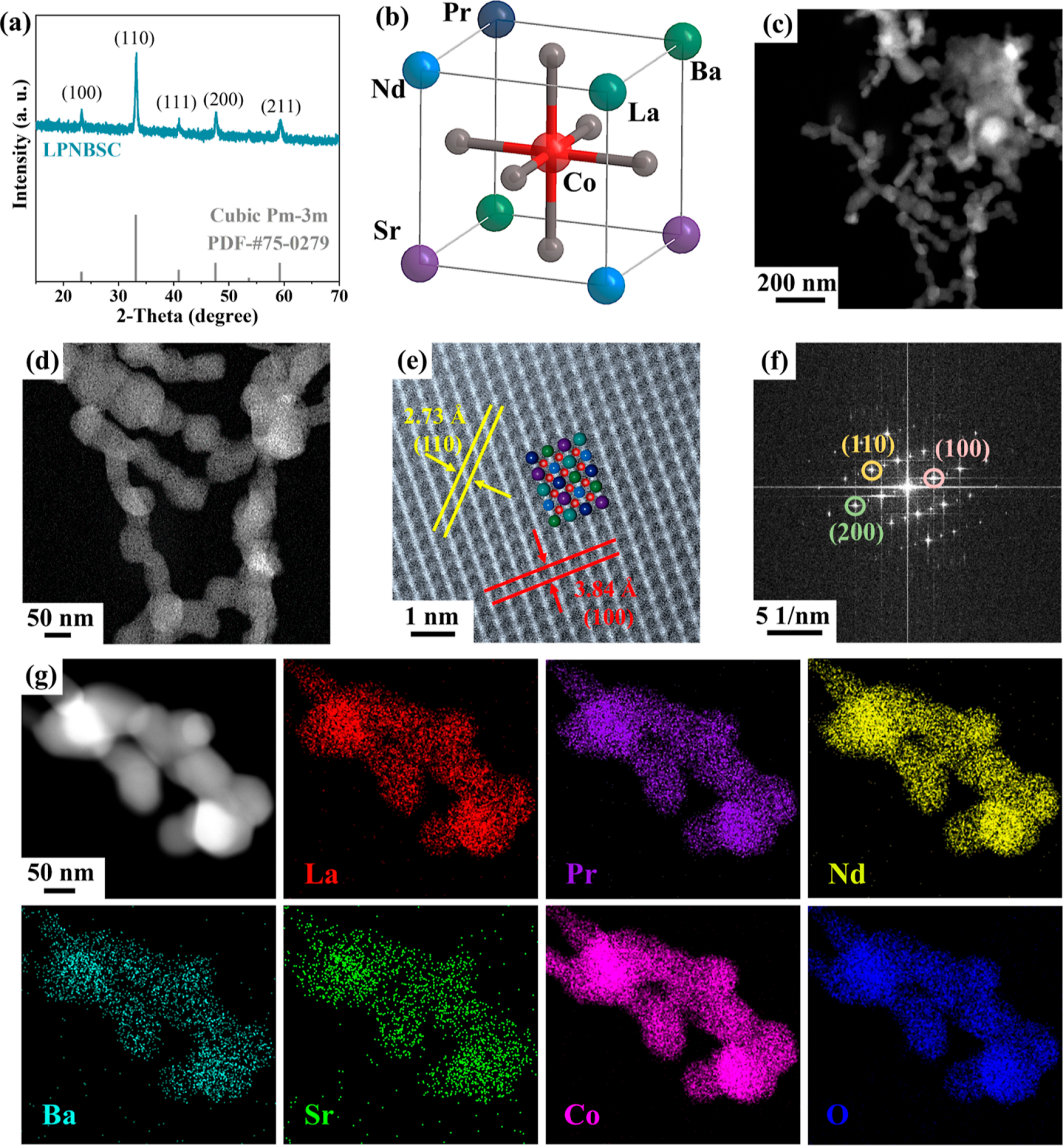
Morphological and structural characterizations of LPNBSC. (a) XRD patterns of LPNBSC. (b) Schematic representation of the LPNBSC perovskite structure. (c,d) Low-resolution and (e) atom-resolved HAADF-STEM images with (f) corresponding FFT patterns along the [100] zone axis. (g) HAADF-STEM and the corresponding elemental mappings of LPNBSC.

The LEPO LaCoO_3_ was synthesized using a hydrothermal method. Then, the HEPO with the composition of (La_0.2_Pr_0.2_Nd_0.2_Ba_0.2_Sr_0.2_)CoO_3-δ_ (LPNBSC) was obtained by the same method (see the experimental details in the Supporting Information). Powder X-ray diffraction (XRD) patterns confirm that LaCoO_3_ adopts a single-phase hexagonal perovskite structure (Figure S1). The XRD patterns of LPNBSC display prominent peaks at 23.3, 33.2, 41.0, 47.6, and 59.3°, corresponding to the (100), (110), (111), (200), and (211) planes of a high-symmetry cubic phase (*Pm*-3*m*) (Figure 2a). This phase transformation induced by substituting La atoms with Pr, Nd, Ba, and Sr suggests that increased entropy can alleviate lattice strain and lead to enhanced structural stability.^40^Figure 2b illustrates the ideal perovskite structure with cubic symmetry, featuring five larger metal elements La/Pr/Nd/Ba/Sr as A-site cations and Co as B-site cations. Characterization by high-angle annular dark-field scanning transmission electron microscopy (HAADF-STEM) reveals a near-spherical morphology of LPNBSC with an average particle size of 50–70 nm (Figure 2c,d). The atom-resolved HAADF-STEM image in Figure 2e shows the LPNBSC structure viewed along the [100] direction, where two types of atomic columns are distinguishable due to differences in their average atomic numbers. The measured interplanar spacings of 3.84 and 2.73 Å are indexed to the (100) and (110) planes of a cubic perovskite.^41−43^ The corresponding fast Fourier transform (FFT) pattern (Figure 2f) displays (100), (110), and (200) reflections in the reciprocal space, indicating a well-formed crystal structure, consistent with XRD analysis. The HAADF-STEM image and its corresponding energy-dispersive X-ray (EDX) elemental mapping analysis display uniform distributions of La, Pr, Nd, Ba, Sr, Co, and O elements across the LPNBSC nanoparticles (Figure 2g). This again confirms the presence of a single-crystal phase. Similarly, the HAADF-STEM image and EDX elemental mappings of LaCoO_3_ were also fully characterized (Figure S2).

**Figure 3:**
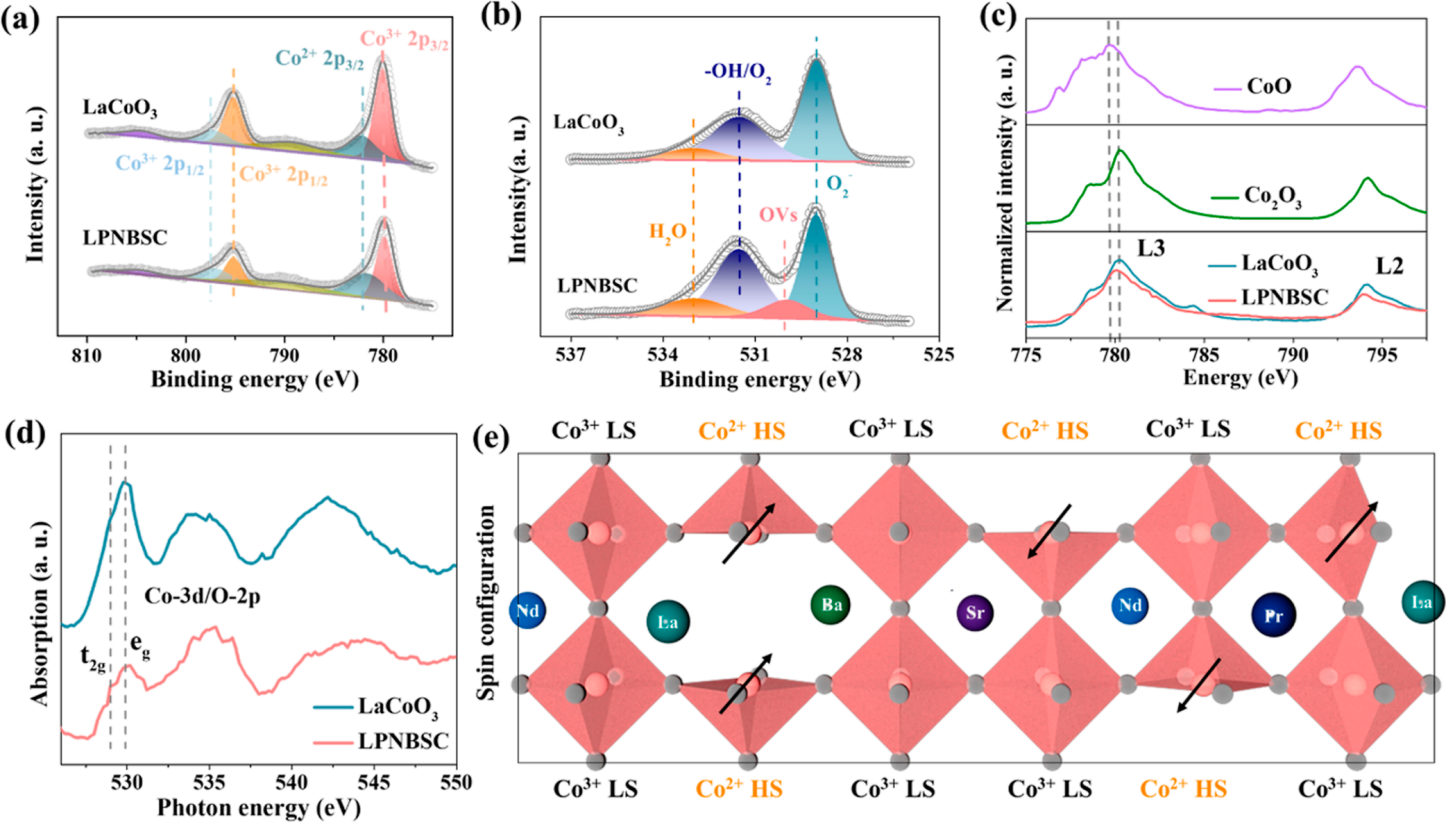
Electronic structure characterization of LaCoO_3_ and LPNBSC. (a) Co 2p spectra. (b) O 1s spectra. (c) Co L-edge and (d) O k-edge XANES spectra. (e) Schematic illustration of spin configuration of Co in LPNBSC.

Then, X-ray photoelectron spectroscopy (XPS) and X-ray absorption near-edge spectroscopy (XANES) were performed to investigate the subtle change in the electronic states of LPNBSC in comparison to LaCoO_3_. The high-resolution Co 2p XPS spectra indicate the coexistence of Co^3+^ and Co^2+^ species in both LaCoO_3_ and LPNBSC (Figure 3a).^42,44^ However, a higher proportion of Co^2+^ was found in LPNBSC compared to that of LaCoO_3_ according to the intensity ratio of the split peaks of Co^2+^ and Co^3+^ (LPNBSC, 0.83 vs LaCoO_3_, 0.39). Compared to LaCoO_3_, the high-resolution O 1s spectrum of LPNBSC shows a new peak at 530.0 eV, assigned to oxygen vacancies (OVs) (Figure 3b).^45−47^ Furthermore, the electron paramagnetic resonance (EPR) spectra reveal that the LPNBSC exhibits a significantly stronger EPR signal at *g* = 2.004 compared to that of LaCoO_3_ (Figure S3), providing additional evidence of the higher concentration of defects in LPNBSC. These OVs increase the content of Co^2+^ and achieve charge balance. The Co L-edge XANES spectra of materials were characterized by the Co 2p → 3d transition and split into L3-edge and L2-edge due to spin–orbit coupling.^48,49^ The Co L3-edges in LaCoO_3_ and LPNBSC are positioned between those in CoO and Co_2_O_3_, confirming the presence of mixed Co^2+^ and Co^3+^ states (Figure 3c). Additionally, a slight shift in the Co L3-edge position in LPNBSC (∼0.2 eV toward lower energy) suggests a decrease in the oxidation state of Co compared to LaCoO_3_. Moreover, the normalized intensity and shape of the Co L3-edge spectrum, especially at lower photon energy, are influenced by the multiple structures arising from Co 3d–3d interactions and the hybridization of Co 3d orbitals with ligand O 2p orbitals. These features provide insights into spin states and reveal the existence of unoccupied Co e_g_ orbitals.^50,51^ The observed decrease in the normalized intensity of LPNBSC suggests partial filling of the e_g_ orbital, indicative of a high spin state in the Co species. The O *K*-edge XANES offers additional evidence for the change in the spin state of Co according to the electron redistribution between e_g_ and t_2g_ of the Co 3d orbitals. The features between 526.5 and 531.5 eV are attributed to the hybridization between unoccupied O 2p states and Co 3d orbitals (Figure 3d).^52^ In LaCoO_3_, a single peak at 530 eV is observed, indicating fully empty e_g_ states. In contrast, a new peak emerges at 529 eV in LPNBSC, accompanied by a reduction in the peak intensity at 530 eV. This shift suggests that electron transfer occurs from t_2g_ orbitals to e_g_ orbitals. This electronic redistribution between the e_g_ and t_2g_ levels creates partially unoccupied t_2g_ states in the Co ions, providing experimental evidence for the spin-state transition induced by A-site high-entropy management. In conclusion, the LS state of Co^3+^ in LaCoO_3_ is associated with a highly symmetrical [CoO_6_] octahedron structure (Figure S4). However, the presence of a large number of OVs in LPNBSC leads to the formation of a high concentration of [CoO_5_] structural motifs, which induces the spin polarization of the Co species (Figure 3e). The increased overlaps and occupied states near the Fermi level (*E*_F_) are primarily ascribed to the Co d and O p hybridized orbitals in LPNBSC, thus facilitating the evolution of the Co spin state from LS (e_g_ = 0) to HS (e_g_ = 1).

**Figure 4:**
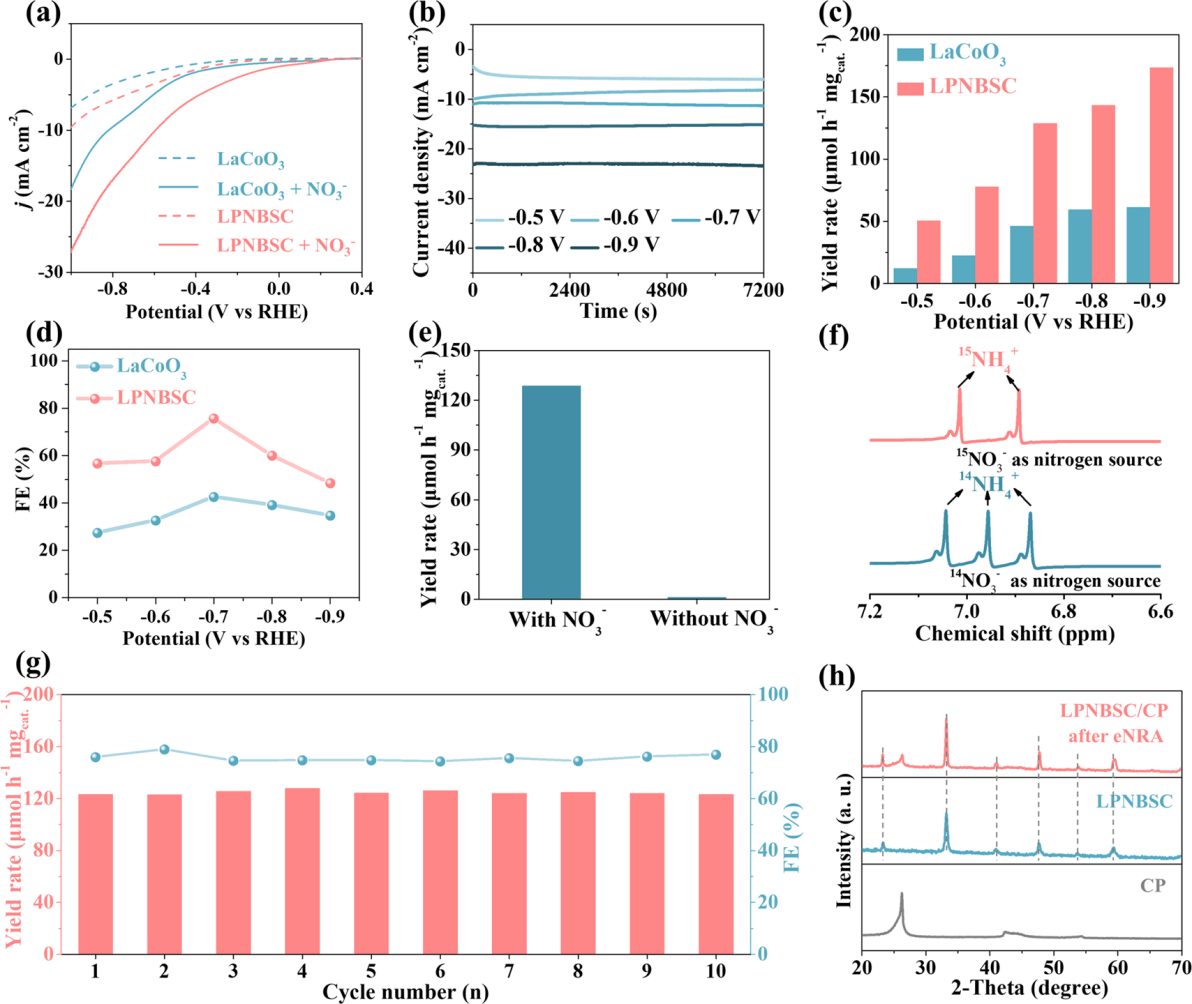
Evaluation of the electrocatalytic eNRA performance of LaCoO_3_ and LPNBSC. (a) LSV curves of both materials in a K_2_SO_4_ electrolyte with and without NO_3_^–^. (b) *I*–*t* curves of LPNBSC under various reduction potentials. (c) NH_3_ yield rates and (d) FE values of materials. (e) NH_3_ yield rates over LPNBSC in the K_2_SO_4_ electrolyte with and without NO_3_^–^. (f) ^1^H NMR spectra of generated ^14^NH_4_^+^ and ^15^NH_4_^+^ when using electrolytes with ^14^NO_3_^–^ and ^15^NO_3_^–^, respectively. (g) Cycling tests of LPNBSC at −0.7 V vs RHE. (h) XRD patterns of fresh and used LPNBSC.

The eNRA performance of LaCoO_3_ and LPNBSC was evaluated using a standard three-electrode system with a solution containing a 0.05 M K_2_SO_4_ electrolyte and 0.1 M NO_3_^–^. As shown in Figure 4a, both materials demonstrate increased current densities shown in linear sweep voltammetry (LSV) curves after addition of NO_3_^–^ to the electrolyte, indicating that both LaCoO_3_ and LPNBSC are active for eNRA. Compared to LaCoO_3_, which exhibites poor activity, LPNBSC displays significantly improved performance. To further investigate this, chronoamperometry (*I*–*t*) tests were conducted for 2 h at various potentials ranging from −0.5 to −0.9 V (Figure 4b). The generated NH_3_ product was quantified using ion chromatography (IC, Figure S5). NH_3_ production becomes detectable at a potential of –0.5 V, with yields increasing as the potential becomes more negative. Specifically, LPNBSC reaches an impressive NH_3_ yield rate of 129 μmol h^–1^ mg_cat._^–1^ at −0.7 V, which is ∼3 times that of LaCoO_3_ (46 μmol h^–1^ mg_cat._^–1^) (Figure 4c). Additionally, the FE of LPNBSC peaks at 76% at this potential, which is ∼2 times that of LaCoO_3_ (42%) (Figure 4d). At reduction potentials above −0.7 V, the insufficient driving force leads to the desorption of intermediates, hindering their further conversion to NH_3_ and consequently lowering the FE. Below −0.7 V, the intensified hydrogen evolution reaction reduces the FE as well. As a result, both LaCoO_3_ and LPNBSC display volcanic trends, with peak performance observed at −0.7 V.^13^ Perovskite oxides typically fail to demonstrate satisfying eNRA activity due to intrinsic limitations, such as low electronic conductivity and a restricted number of surface-active sites resulting from the saturated coordination of A-site and B-site cations with oxygen atoms.^53,54^ However, the eNRA performance of LPNBSC is much higher than that of previously reported perovskites such as LaFe_0.9_Cu_0.1_O_3-δ_ (FE: 47.8%), La_2_Cu_0.8_Ni_0.2_O_4_ (FE: 45.7%), and La_2_CuO_4_ (FE: 29.3%) (Table S1). To confirm the accuracy of our measurements, we also quantified the NH_3_ yield using the indophenol method (Figure S6). The results are consistent with those obtained by the IC method (Figure S7). Control experiments confirm that NH_3_ production is negligible in the absence of NO_3_^–^ (Figure 4e). Moreover, isotope labeling experiments were conducted to identify the nitrogen source of NH_3_ produced during the eNRA process. The ^1^H NMR spectrum shows doublets at δ = 6.89 and 7.02 ppm for ^15^NH_4_^+^ when ^15^NO_3_^–^ is used as the nitrogen source, whereas triplets at δ = 6.87, 6.95, and 7.04 ppm are observed for ^14^NH_4_^+^ when the nitrogen source is ^14^NO_3_^–^ (Figure 4f). This confirms that the formation of NH_3_ indeed originates from the NO_3_^–^ anion. Note that the small peaks adjacent to each main peak are due to spin–spin coupling between the nitrogen nuclei and the protons in NH_4_^+^.^55^ The durability of LPNBSC was evaluated through 10 consecutive electrolysis cycles at −0.7 V (Figure 4g). The NH_3_ yield rate and FE remain stable across all cycles. XRD analysis proves that the crystal structure of the used LPNBSC remains intact (Figure 4h). EDX mappings further demonstrate that no element segregation occurrs after the eNRA reaction (Figure S8). These results highlight the structural stability and persistent performance of HEPO. Additionally, Nyquist plots reveal that LPNBSC has lower charge-transfer resistance and faster electron-transfer rates compared to LaCoO_3_ (Figure S9). The electrochemically active surface area (ECSA) is a crucial parameter for assessing the effectiveness of active sites in electrochemical reactions.^56−58^ The ECSAs of LaCoO_3_ and LPNBSC were estimated by measuring the double-layer capacitance (*C*_dl_) using cyclic voltammetry curves (Figures S10 and S11). The *C*_dl_ value of LPNBSC is comparable to that of LaCoO_3_. These results indicate that the better eNRA performance of LPNBSC can be solely attributed to its higher intrinsic activity.

**Figure 5:**
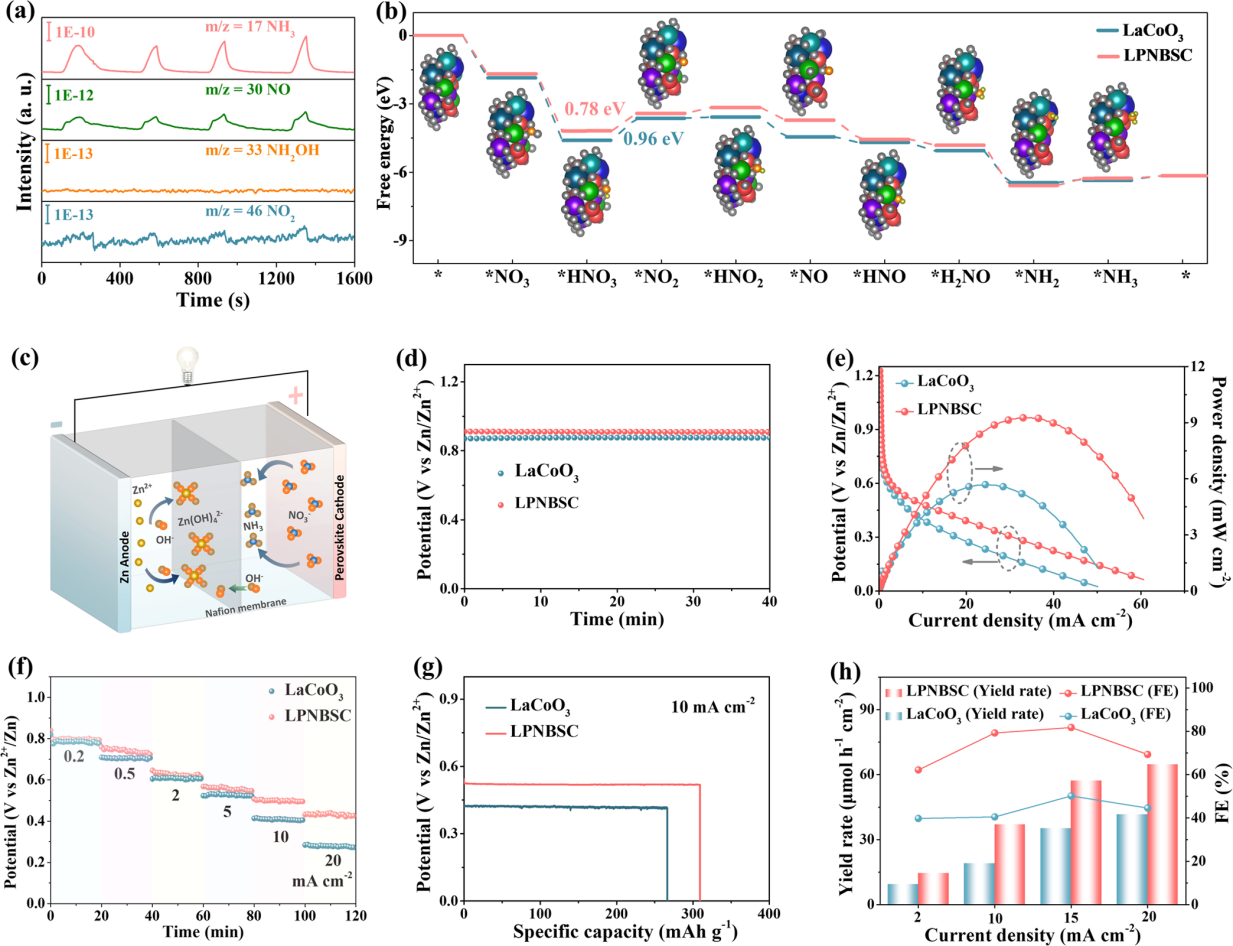
LPNBSC catalyst for mechanistically studying the eNRA process and its application in a Zn–NO_3_^–^ battery. (a) DEMS measurements of LPNBSC for eNRA. (b) Free energy profiles for eNRA catalysis on LaCoO_3_ and LPNBSC models. (c) Schematic illustration of the Zn–NO_3_^–^ battery setup. (d) Open-circuit potentials (OCPs), (e) polarization and power density curves, (f) discharging tests at various current densities, (g) voltage profiles versus specific capacities, and (h) NH_3_ yield rates, corresponding FE values at various current densities of LaCoO_3_-based and LPNBSC-based Zn–NO_3_^–^ batteries.

To obtain insights into the catalytic mechanism of eNRA using LPNBSC, we used online DEMS to detect molecular intermediates and products. Signals for mass-to-charge ratios (*m*/*z*) of 30, 44, and 17 corresponding to NO, NO_2_, and NH_3_, respectively, were observed throughout four continuous test cycles (Figure 5a). These findings confirm that NO and NO_2_ are intermediates during the eNRA process. Notably, we did not detect an *m*/*z* signal of 33, which corresponds to NH_2_OH, excluding NH_2_OH as a byproduct. To study the possible reaction pathway of eNRA, we built and optimized models of the (100) surfaces of HEPO and LaCoO_3_ (Figure S12). The optimized HEPO model features [CoO_5_] structural motifs (Figure S12a). We performed a charge population analysis to evaluate the formal charge state of Co. In the [CoO_5_] structural motif of LPNBSC, the Co center exhibits a lower formal charge (0.35) compared to the Co center in the [CoO_6_] motif of LaCoO_3_ (0.44) (Figure S13). This suggests a reduced charge density around the Co sites in LPNBSC, likely due to the presence of OVs. These findings are consistent with the XPS results. Furthermore, in LaCoO_3_, the Co atom in the [CoO_6_] structural motifs demonstrates nearly equal spin-up and spin-down contributions near the *E*_F_, indicating paired electron states (Figure S14). In contrast, the Co atom in the [CoO_5_] structural motifs of LPNBSC shows a significant disparity between spin-up and spin-down components near *E*_F_, reflecting the presence of unpaired electrons in the Co-3d orbitals.^59^ These results align well with the experimental observation where the formation of OVs in LPNBSC introduces unpaired electronic states near *E*_F_. Then, different steps in the whole eNRA process were studied with the corresponding free energy profiles, as displayed in Figure 5b. The eNRA process begins with the adsorption of NO_3_^–^ on the catalyst surfaces. On the HEPO surface, one O atom from NO_3_^–^ binds to the HS Co site. Subsequently, *NO_3_ reacts with a proton (H^+^) and an electron (e^–^) to form *HNO_3_, a process that is energetically favorable. The next step involves the removal of one exposed O atom from *HNO_3_ to produce *NO_2_ and H_2_O. This step has energy barriers of 0.96 and 0.78 eV for LaCoO_3_ and LPNBSC, respectively, indicating that *HNO_3_ + H^+^ + e^–^ → *NO_2_ + H_2_O is the thermodynamic rate-determining step (RDS) for the eNRA process. Then, converting *NO_2_ to *HNO_2_ requires overcoming small energy barriers of 0.04 and 0.25 eV for LaCoO_3_ and LPNBSC, respectively. *HNO_2_ then spontaneously loses another O atom to form *NO. Following this, *NO undergoes stepwise hydrogenation to generate intermediates of *HNO and *H_2_NO. *H_2_NO then undergoes a third deoxygenation step to produce *NH_2_. Finally, *NH_2_ is readily hydrogenated and desorbed from the catalyst surface to form NH_3_. In conclusion, the high-entropy management in the A-site of the perovskite structure facilitates the generation of abundant OVs, thereby transforming the Co-active center from the LS state to the HS state. This transformation lowers the energy barrier for the RDS of *HNO_3_ deoxygenation, accelerates the production of *NO_2_, and enhances both the catalytic activity and FE of the HEPO electrode for eNRA. Furthermore, the free energy profile for eNRA catalysis within the LPNBSC model at an optimized potential of −0.7 V vs RHE is presented in Figure S15. At this potential, the free energy barriers for all reaction steps are low, allowing each step to proceed efficiently.

Zinc-nitrate (Zn–NO_3_^–^) batteries offer the dual advantage of generating clean energy while recycling NO_3_^–^ from wastewater into valuable NH_3_.^56,60^ To explore the potential of LPNBSC in such applications, we tested the homemade Zn–NO_3_^–^ battery using LPNBSC as the cathode and Zn foil as the anode (Figure 5c). The LPNBSC-based Zn–NO_3_^–^ battery maintains a stable OCP of 0.9 V vs Zn^2+^/Zn (Figure 5d). This value is higher than that of LaCoO_3_ (0.87 V). The discharge polarization and power density curves provide evidence that LPNBSC deliveres a current density of up to 15 mA cm^–2^ at 0.45 V (Figure 5e). The peak power density reaches 9.3 mW cm^–2^ at 0.29 V, which is significantly higher than 5.7 mW cm^–2^ at 0.23 V observed for the LaCoO_3_-based battery. Additionally, the LPNBSC-based battery demonstrates a high potential at various discharge current densities (Figure 5f). The high specific capacity (309 mAh g^–1^ at 10 mA cm^–2^) indicates a good overall performance of LPNBSC (Figure 5g). Remarkably, the LPNBSC-based Zn–NO_3_^–^ battery achieves a maximum FE of 82% at 15 mA cm^–2^, with the NH_3_ yield rate as high as 57 μmol h^–1^ cm^–2^, which is 1.6 times that of the LaCoO_3_-based battery (Figure 5h). These results highlight the effectiveness of LPNBSC in enhancing the performance of Zn–NO_3_^–^ batteries, making it a promising material for energy output and NH_3_ production.

## Conclusions

In summary, we have developed an A-site high-entropy management scheme to synthesize HEPO (La_0.2_Pr_0.2_Nd_0.2_Ba_0.2_Sr_0.2_)CoO_3-δ_. The introduction of diverse A-site cations resulted in a higher concentration of OVs and HS Co-active sites compared to that of the traditional LEPO LaCoO_3_. DFT calculations revealed that the [CoO_5_] structural motifs in the A-site high-entropy perovskite can enhance NO_3_^–^ activation and lower the thermodynamic energy barrier of the rate-limiting step (*HNO_3_ + H^+^ + e^–^ → *NO_2_ + H_2_O). When applied as the cathode in a Zn–NO_3_^–^ battery, LPNBSC demonstrated superior performance compared to LEPO LaCoO_3_ and reported perovskite-based catalysts. Future work should focus on understanding the long-term stability of these materials under real-world applications, particularly addressing potential issues related to cation segregation and phase stability. Overall, this study opens up new avenues for the design of advanced electrocatalysts and highlights the transformative potential of high-entropy strategies in the fields of energy conversion and environmental sustainability.

## Supporting Information Available

Experimental section including preparation of materials, characterizations, electrochemical measurements, charge population, and PDOS analysis of Co sites in LPNBSC and LaCoO_3_; details of free energy calculations for eNRA reaction steps; EPR spectra showing defect features; and computational details, including DFT models and parameters (PDF)
