# Supplementary material for: Improved Ammonia Synthesis and Energy Output from Zinc-Nitrate Batteries by Spin-State Regulation in Perovskite Oxides
Source: J Am Chem Soc. 2025 Jan 16;147(4):3119–28. doi: 10.1021/jacs.4c12240 (PMC11783523; doi:10.1021/jacs.4c12240)
Supplement: Supplementary file 1 — ja4c12240_si_001.pdf [file ja4c12240_si_001.pdf]

Improved Ammonia Synthesis and Energy Output from Zinc-Nitrate Batteries by Spin-State Regulation in Perovskite Oxides

Hele Guo[1,2○], Yazhou Zhou[3,4○], Kaibin Chu[1,2○], Xueying Cao[5○], Jingjing Qin[1], Nan Zhang[1], Maarten B. J. Roeffaers[6], Radek Zbořil[4,7], Johan Hofkens[2,3], Klaus Müllen[3*], Feili Lai[2,8*], Tianxi Liu[1*]

[1]*The Key Laboratory of Synthetic and Biological Colloids, Ministry of Education, School of Chemical and Material Engineering, Jiangnan University, Wuxi 214122, China.*

[2]*Department of Chemistry, KU Leuven, Leuven 3001, Belgium*

[3]*Max Planck Institute for Polymer Research, Mainz 55128, Germany.*

[4]*Nanotechnology Centre, Centre for Energy and Environmental Technologies (CEET), VŠB—Technical University of Ostrava, Ostrava-Poruba 708 00, Czech Republic.*

[5]*Key Laboratory for Colloid and Interface Chemistry, Ministry of Education, School of Chemistry and Chemical Engineering, Shandong University, Jinan 250100, P. R. China.*

[6]*cMACS, Department of Microbial and Molecular Systems, KU Leuven, Leuven 3001, Belgium.*

[7]*Regional Centre of Advanced Technologies and Materials, Czech Advanced Technology and Research Institute (CATRIN), Palacký University Olomouc, Olomouc 779 00, Czech Republic.*

[8]*State Key Laboratory of Metal Matrix Composites, School of Materials Science and Engineering, Shanghai Jiao Tong University, Shanghai 200240, P. R. China.*

[○]*These authors contributed equally to this work.*

Experimental section

Materials

Analytical reagents used in the experiments were bought from Sigma Aldrich: $La(NO_3)_3{\cdot}6H_2O$, $Pr(NO_3)_3{\cdot}6H_2O$, $Nd(NO_3)_3{\cdot}6H_2O$, $Ba(NO_3)_2$, $Sr(NO_3)_2$, $Co(NO_3)_2{\cdot}6H_2O$, KNO_3, $K^{15}NO_3$, K_2SO_4, KCl, KOH, NaOH, Nafion (5 wt.%), and ethanol. Deionized (DI) water was used throughout the experiments.

Preparation of $LaCoO_3$ and $(La_{0.2}Pr_{0.2}Nd_{0.2}Ba_{0.2}Sr_{0.2})CoO_{3-\delta}$ (LPNBSC)

$LaCoO_3$ and LPNBSC were prepared by a facile hydrothermal method. Analytically pure nitrate reagents including $La(NO_3)_3{\cdot}6H_2O$, $Nd(NO_3)_3{\cdot}6H_2O$, $Pr(NO_3)_3{\cdot}6H_2O$, $Ba(NO_3)_2$, $Sr(NO_3)_2$, and $Co(NO_3)_2{\cdot}6H_2O$ were used as precursors. By taking LPNBSC as an example, stoichiometric amounts of $La(NO_3)_3{\cdot}6H_2O$, $Nd(NO_3)_3{\cdot}6H_2O$, $Pr(NO_3)_3{\cdot}6H_2O$, $Ba(NO_3)_2$, $Sr(NO_3)_2$, and $Co(NO_3)_2{\cdot}6H_2O$ with the ratio of La: Nd: Pr: Ba: Sr: Co = 0.4: 0.4: 0.4: 0.4: 0.4: 2 were dissolved in 30 mL deionized water and stirred at 700 rpm to form a clear solution. 30 mL of 0.4 M NaOH solution was then added slowly. After stirring for 15 min, the suspension was transferred to a 100 mL Teflon-lined stainless-steel autoclave and heated at 180 °C for 10 h before cooling to room temperature. The resultant precipitate was washed with deionized water and ethanol three times and then dried at 70 °C for 5 h. The obtained powder was calcined at 750 °C in air for 5 h, to obtain the perovskite structure. The $LaCoO_3$ was also prepared by the same method with a La/Co ratio of 2:2.

Characterizations

The morphology of powder samples was evaluated by high-angle annular dark field scanning transmission electron microscopy (HAADF-STEM, EM-ARM300F). X-ray diffraction (XRD) patterns were collected on a Bruker D8 X-ray diffractometer with a Cu Kα X-ray source (λ = 1.5418 Å). High-resolution transmission electron microscopy (HRTEM) was performed using a JEOL JEM-2100Plus electron microscope. X-ray photoelectron spectroscopy (XPS) measurements were carried out with an AXIS Supra. Ultraviolet-visible (UV-Vis) absorbance spectra were measured on a TU1900 spectrophotometer. ^1H nuclear magnetic resonance (^1H NMR) was performed on a Bruker Avance DRX 600 spectrometer. Synchrotron-based X-ray absorption spectra (XAS) of Co L-edge and O K-edge were conducted at the BL14W1 beamline of Shanghai Synchrotron Radiation Facility (SSRF). Online differential

electrochemical mass spectrometry (DEMS, QAS 100) experiments were performed on the QAS 100 instrument.

Electrochemical measurements

Before electrochemical tests, the Nafion membrane was protonated by boiling in 5% H_2O_2 aqueous solution for 1 h, then in water for an additional hour, followed by 3 h in a 0.5 M H_2SO_4, and finally for 6 h in water. The electrochemical activity for the electrocatalytic nitrate reduction to ammonia (eNRA) in 0.05 M K_2SO_4 solution with 0.1 M KNO_3 was performed by CHI 660D electrochemical workstation in a standard three-electrode system, with the Ag/AgCl electrode as the reference electrode, a graphite rod as the counter electrode, and the catalyst loaded on a carbon paper (CP) electrode as the working electrode. The electrolyte was degassed by bubbling Ar for 30 min before the eNRA measurements. In a typical procedure for the fabrication of the working electrode, 6 mg of catalyst and 60 μL of a 5 wt% Nafion solution were dispersed in 940 μL of absolute ethyl alcohol. The mixture was sonicated to achieve a homogeneous ink. Subsequently, 90 μL of the prepared ink was dropped to a CP electrode with dimensions of 1 cm × 1 cm. The electrode was then dried in an Ar atmosphere at 80 °C for 1 h. All potentials were converted to the potential of the relatively reversible hydrogen electrode (RHE) by the formula (E (*vs.* RHE) = E (*vs.* Ag/AgCl) + 0.197 + 0.059 × pH). Linear sweep voltammetry (LSV) curves were scanned in the range of -1.0 to 0.4 V. The cyclic voltammetry curves were scanned under a potential window between 0.67 and 0.77 V. Electrochemical impedance spectroscopy (EIS) was measured at -0.7 V from 0.1 Hz to 100 kHz.

The electrochemical performance of the homemade Zn-NO_3^- battery in a two-electrode system was determined in an H-type cell. A piece of (1×1 cm^2) $LaCoO_3$ or LPNBSC cathode in 0.5 M NO_3^-/2 M KCl electrolyte and Zn foil anode (1×1 cm^2) in 2 M KOH were separated by a Nafion 117 membrane. All battery data were recorded with a CHI 660D electrochemical workstation.

Determination and quantitation of NH_3

Determination of NH_3 using the ion chromatography method: The pristine blank electrolyte and cathodic electrolyte were diluted 50 times using deionized water. A series of standard solutions of NH_4Cl (0.1, 0.25, 0.5, 1, 2.5, 5, 10 ppm) in diluted blank electrolyte were prepared

to establish the NH_4^+ concentration-peak area calibration curves. The concentration of NH_3 produced in the eNRA was determined according to the calibration curve and the measured peak area.

Determination of NH_3 using the indophenol blue method: The concentration of produced NH_3 in the electrolytes were also spectrophotometrically determined by the indophenol blue method. In detail, 2 mL of 1 M NaOH solution containing salicylic acid (5 wt.%) and sodium citrate (5 wt.%) was added. Subsequently, 1 mL of NaClO solution (0.05 M) and 0.2 mL of sodium nitroferricyanide solution (1 wt.%) were added. Absorbance measurements were performed from 550 to 750 nm. The concentration-absorbance (at 655 nm) curve was calibrated using a standard NH_4^+ solution at various concentrations. The concentration of NH_4^+ in the electrolyte is obtained by diluting a certain number of times on this basis.

^{15}N Isotopic Tracing experiments

$^{15}NO_3^-$ (99 atom%, ≥99%) was used to trace the nitrogen source of the eNRA. After 2 h of eNRA in the 0.05 M K_2SO_4 electrolyte containing 0.1 M $^{15}NO_3^-$, the produced $^{15}NH_4^+$ in the electrolyte was detected using ^1H NMR (600 MHz) spectroscopy.

Online DEMS measurements

The 0.05 M K_2SO_4 electrolyte with 0.1 M KNO_3 was kept flowing into a homemade electrochemical cell through a peristaltic pump. Ar gas was bubbled into the electrolyte continuously before and during the DEMS measurements. The glassy carbon electrode coated with the LPNBSC catalyst, the Pt wire, and the Ag/AgCl electrode were used as the working electrode, counter electrode, and reference electrode, respectively. The LSV technique was employed from 0 to -1.0 V at a scan rate of 10 mV s^{-1} until the baseline stabilized. Then, the corresponding mass spectral signals appeared. After the electrochemical test ended and the mass signal returned to baseline, the next cycle started using the same conditions to avoid accidental errors. After four cycles, the experiment ended.

Density functional theory (DFT) calculations

The DFT calculations were conducted using DMOL3 in Materials Studio, employing the plane-wave pseudopotential method for the first-principles-based quantum mechanics simulations.[1-3] In our models, as reported in the previous work,[4] we used the computational hydrogen electrode (CHE) model for the calculations. Herein, neither

implicit nor explicit solvents were included. The applied electrochemical potentials were set to 0 and -0.7 V by default. The temperature was fixed at 298.15 K. A vacuum slab of 15 Å was employed to avoid the interaction of the periodic boundary conditions. A Γ-centred Monkhorst-Pack mesh with 2 × 2 × 1 K-points was used for Brillouin zone integration. During computation, the double numerical plus polarization functions (DNP = 4.4) were used as the basis set. The Kohn-Sham self-consistent field calculations were performed with convergence tolerance of 1×10^{-6} Hartree on the total energy.

For the Gibbs free energy (ΔG) calculations, the zero-point energy (ΔZPE) and entropy corrections are considered in the absorption energy calculations.

$$\Delta G = \Delta E + \Delta ZPE - T\Delta S$$

In which ΔE is the difference in electronic energy in the ground state obtained from self-consistent calculation, T is the temperature, ΔS is the entropy difference. The entropies of molecules in the gas phase were taken from the NIST database.

Calculation of the NH_3 yield rate, the Faradaic efficiency (FE) of NH_3

NH_3 yield rate was calculated using the following equation:

$$NH_3 \text{ yield rate} = [NH_4^+] \times V / (m_{cat.} \times t)$$

FE was calculated according to the following equation:

$$FE = 8 \times F \times [NH_4^+] \times V / (17 \times Q)$$

Where $[NH_4^+]$ is the measured NH_4^+ concentration, V is the volume of the cathodic reaction electrolyte, t is the potential applied time, $m_{cat.}$ is the mass of catalyst, F is the Faraday constant, and Q is the quantity of applied electricity.

Figures

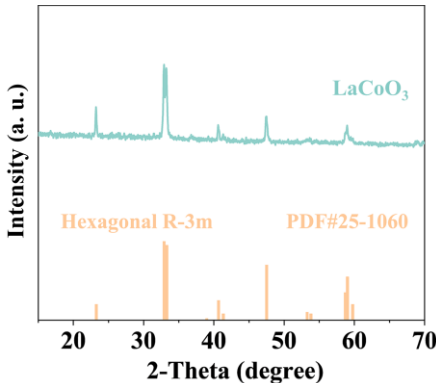

Figure S1. XRD patterns of $LaCoO_3$.

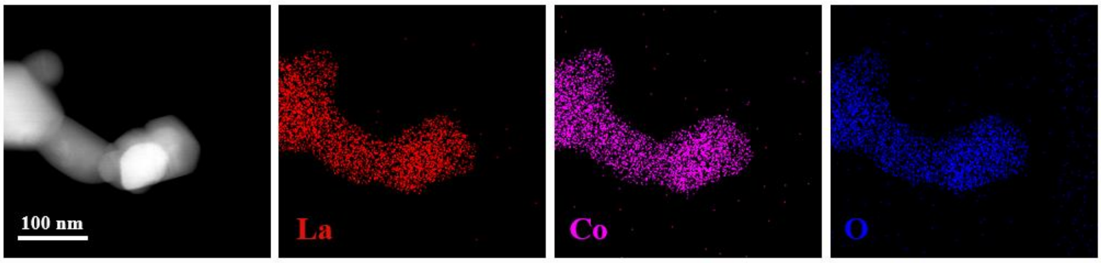

Figure S2. HAADF-STEM and the corresponding elemental mappings of $LaCoO_3$.

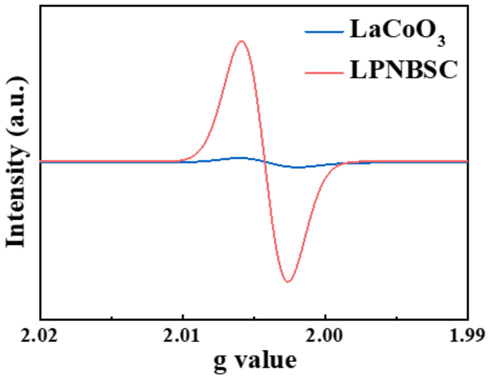

Figure S3. EPR spectra of $LaCoO_3$ and LPNBSC.

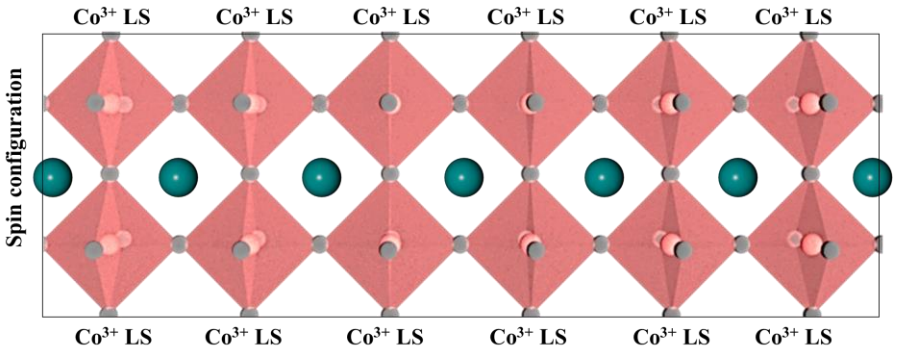

Figure S4. Schematic illustration of spin configuration of Co species in $LaCoO_3$.

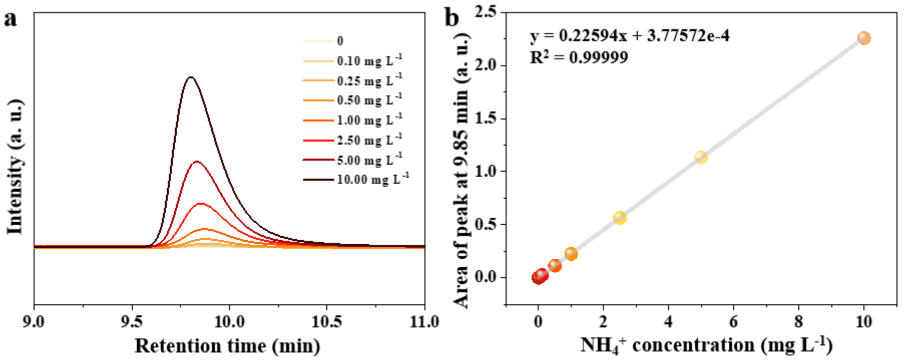

Figure S5. a) Ion chromatograms of NH_4^+ with different concentrations and b) corresponding standard curve.

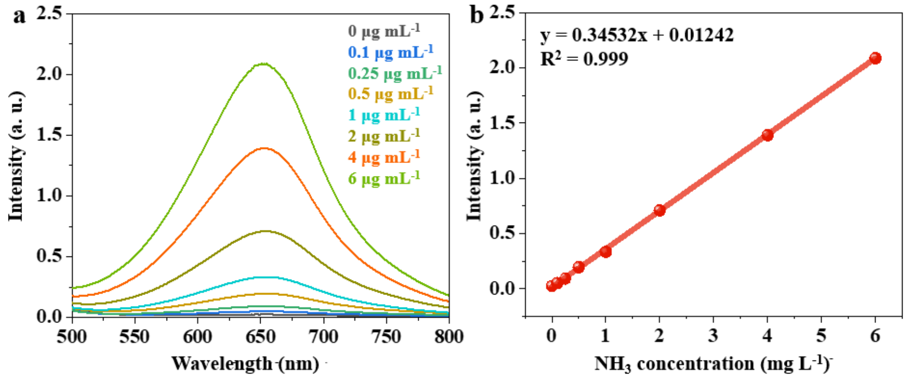

Figure S6. a) UV-vis curves of indophenol assays after incubation for 2 h and b) calibration curve used for estimation of NH_3 concentration.

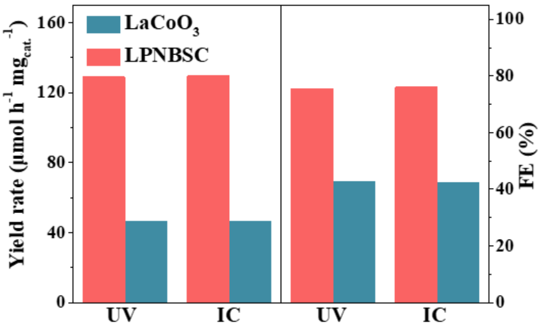

Figure S7. The quantitative comparison of the NH_3 yield rates and FE values achieved by the $LaCoO_3$ and LPNBSC catalysts based on the ion chromatogram and indophenol blue methods.

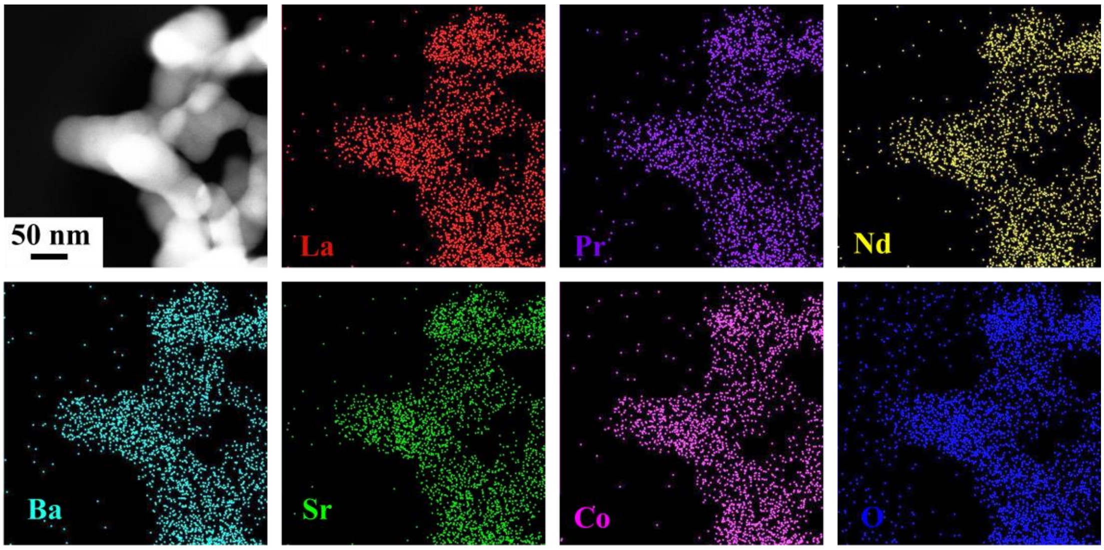

Figure S8. HAADF-STEM and the corresponding elemental mappings of LPNBSC after eNRA process.

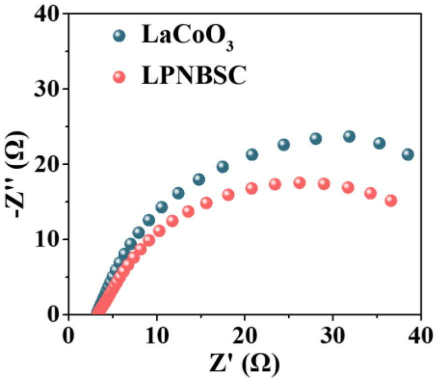

Figure S9. Nyquist plots of the $LaCoO_3$ and LPNBSC catalysts.

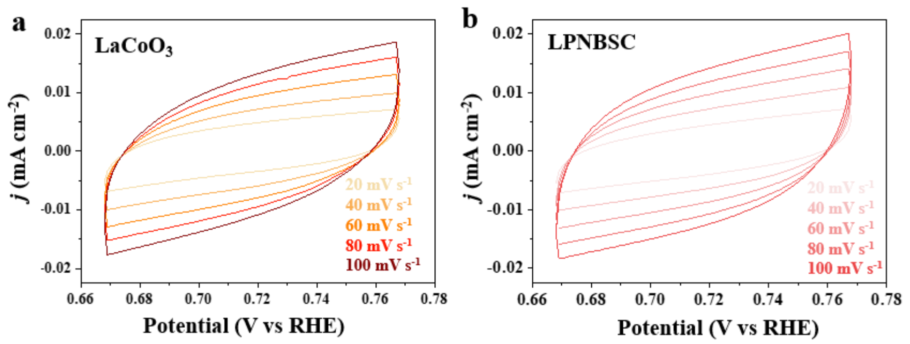

Figure S10. Electrochemical CV curves of a) $LaCoO_3$, b) LPNBSC catalysts recorded at different scanning rates.

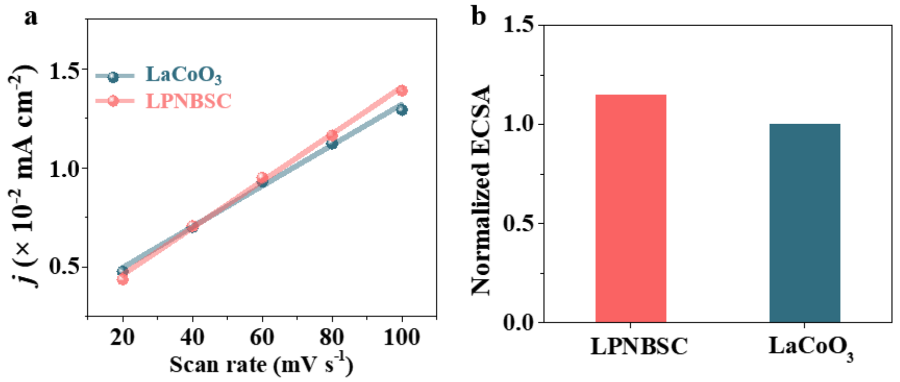

Figure S11. a) The measured capacitive currents are plotted as a function of scan rates. b) Normalized ECSAs of $LaCoO_3$ and LPNBSC catalysts.

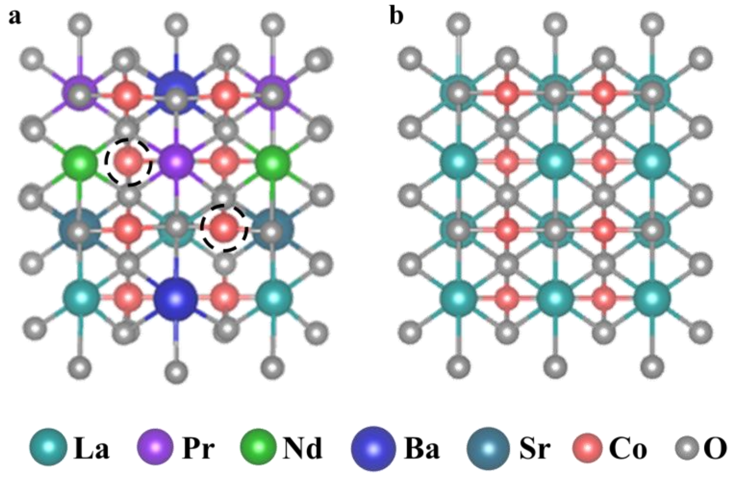

Figure S12. The optimized a) LPNBSC(100) and b) $LaCoO_3$(100) models. The black dashed circles in (a) represent the Co centers in the [CoO_5] structural motifs.

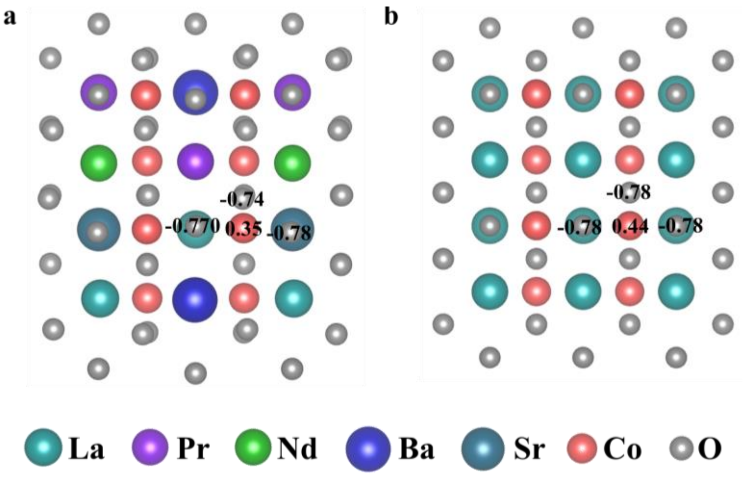

Figure S13. Optimized configuration of a) LPNBSC and b) $LaCoO_3$ models. the numbers represent the formal charges calculated through Mulliken charge analysis.

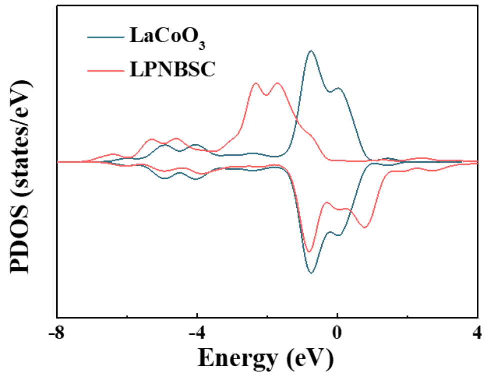

Figure S14. PDOS of Co atoms in the [CoO_6] structural motifs of $LaCoO_3$ and in the [CoO_5] structural motifs of LPNBSC.

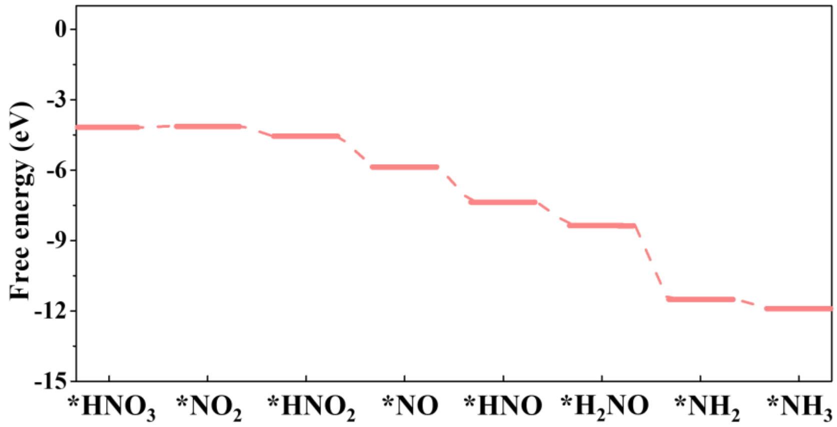

Figure S15. Free energy profile for eNRA on LPNBSC model at the optimized potential of −0.7 V vs. RHE.

Table S1. Comparison of NH_3 selectivity by electrocatalytic NO_3^- reduction.

Electrocatalyst	Electrolyte	FE (%)	NH_3 yield rate (μmol h^{-1} $mg^{-1}_{cat.}$)	Ref.
$LaFe_{0.9}Cu_{0.1}O_{3-\delta}$	50 ppm NO_3^--N + 0.5 M Na_2SO_4	47.8	20.5	5
Fe SACs	0.5 M KNO_3 + 0.1 M K_2SO_4	75.0	460.0 μmol h^{-1} cm^{-2}	6
$La_2Cu_{0.8}Ni_{0.2}O_4$	50 ppm NO_3^--N + 0.5 M Na_2SO_4	45.7	69.9	7
La_2CuO_4	50 ppm NO_3^--N + 0.05 M Na_2SO_4	29.3	—	8
amorphous Ru nanoclusters	500 ppm NO_3^- + 5 mM Cs_2CO_3	80.6	8.5	9
Co_3O_4@NiO	200 ppm NO_3^--N + 0.5 M Na_2SO_4	55.0	6.93	10
Co_3O_4/CF	50 ppm NO_3^- + 0.05 M Na_2SO_4	22.2	16.6	11
Pd-NDs/Zr-MOFs	500 ppm $NaNO_3$ + 0.1 M Na_2SO_4	58.1	287.3	12
O-Cu–PTCDA	500 ppm NO_3^- + 0.1 M PBS	85.9	25.6	13
LPNBSC	0.1 M NO_3^- + 0.05 M K_2SO_4	76	129	This work

References

(1) Perdew, J. P.; Burke, K.; Ernzerhof, M. Generalized Gradient Approximation Made Simple. *Phys. Rev. Lett.* **1996**, *77*, 3865-3868.

(2) Delley, B. An all-electron numerical method for solving the local density functional for polyatomic molecules. *J. Chem. Phys.* **1990**, *92*, 508-517.

(3) Kresse, G.; Furthmüller, Efficiency of ab-initio total energy calculations for metals and semiconductors using a plane-wave basis set. *J. Comput. Mater. Sci.* **1996**, *6*, 15-50.

(4) Nørskov, J. K. Rossmeisl, J. Logadottir, A. Lindqvist, L. Kitchin, J. R. Bligaard, T. Jónsson, H. Origin of the Overpotential for Oxygen Reduction at a Fuel-Cell Cathode. *J. Phys. Chem. B* **2004**, 108, 17886−17892.

(5) Chu, K.; Zong, W.; Xue, G.; Guo, H.; Qin, J.; Zhu, H.; Zhang, N.; Tian, Z.; Dong, H.; Miao, Y. E.; Roeffaers, M. B. J.; Hofkens, J.; Lai, F.; Liu, T. Cation Substitution Strategy for Developing Perovskite Oxide with Rich Oxygen Vacancy-Mediated Charge Redistribution Enables Highly Efficient Nitrate Electroreduction to Ammonia. *J. Am. Chem. Soc.* **2023**, *145*, 21387-21396.

(6) Wu, Z.-Y.; Karamad, M.; Yong, X.; Huang, Q.; Cullen, D. A.; Zhu, P.; Xia, C.; Xiao, Q.; Shakouri, M.; Chen, F.-Y.; Kim, J. Y.; Xia, Y.; Heck, K.; Hu, Y.; Wong, M. S.; Li, Q.; Gates, I.; Siahrostami, S.; Wang, H. Electrochemical ammonia synthesis via nitrate reduction on Fe single atom catalyst. *Nat. Commun.* **2021**, *12*, 2870.

(7) Gong, Z.; Zhong, W.; He, Z.; Jia, C.; Zhou, D.; Zhang, N.; Kang, X.; Chen, Y. Improving electrochemical nitrate reduction activity of layered perovskite oxide La_2CuO_4 via B-site doping. *Catal. Today* **2022**, *402*, 259-265.

(8) Yang, W.-J.; Yang, L.-H.; Peng, H.-J.; Lv, S.-H. Perovskite oxide $LaMO_{3-\delta}$ (M = Fe, Co, Ni and Cu) cathode for efficient electroreduction of nitrate. *Sep. Purif. Technol.* **2022**, *295*, 121278.

(9) Jiang, M.; Tao, A.; Hu, Y.; Wang, L.; Zhang, K.; Song, X.; Yan, W.; Tie, Z.; Jin, Z. Crystalline Modulation Engineering of Ru Nanoclusters for Boosting Ammonia Electrosynthesis from Dinitrogen or Nitrate. *ACS Appl. Mater. Interfaces* **2022,** *14* (15), 17470-17478.

(10) Wang, Y.; Liu, C.; Zhang, B.; Yu, Y. Self-template synthesis of hierarchically structured Co_3O_4@NiO bifunctional electrodes for selective nitrate reduction and tetrahydroisoquinolines semidehydrogenation. *Sci. China Mater.* **2020**, *63*, 2530-2538.

(11) Fu, W.; Du, X.; Su, P.; Zhang, Q.; Zhou, M. Synergistic Effect of Co(III) and Co(II) in a 3D Structured Co_3O_4/Carbon Felt Electrode for Enhanced Electrochemical Nitrate Reduction Reaction. *ACS Appl. Mater. Interfaces* **2021**, *13*, 28348-28358.

(12) Jiang, M.; Su, J.; Song, X.; Zhang, P.; Zhu, M.; Qin, L.; Tie, Z.; Zuo, J.-L.; Jin, Z. Interfacial Reduction Nucleation of Noble Metal Nanodots on Redox-Active Metal–Organic Frameworks for High-Efficiency Electrocatalytic Conversion of Nitrate to Ammonia. *Nano Lett.* **2022**, *22*, 2529-2537.

(13) Chen, G.-F.; Yuan, Y.; Jiang, H.; Ren, S.-Y.; Ding, L.-X.; Ma, L.; Wu, T.; Lu, J.; Wang, H. Electrochemical reduction of nitrate to ammonia via direct eight-electron transfer using a copper–molecular solid catalyst. *Nat. Energy* **2020**, *5*, 605-613.
